# On the control mechanisms of the nitrite level in *Escherichia coli* cells: the mathematical model

**DOI:** 10.1186/s12866-015-0619-x

**Published:** 2016-01-27

**Authors:** Tamara M. Khlebodarova, Nataly A. Ree, Vitaly A. Likhoshvai

**Affiliations:** Institute of Cytology and Genetics SB RAS, Novosibirsk, Russia; Novosibirsk State University, Novosibirsk, Russia

**Keywords:** *Escherichia coli*, Anaerobic respiration, Mathematical model, Gene expression regulation, Nitrite reductase, Nitrite transport, Membrane potential

## Abstract

**Background:**

Due to a high toxicity of nitrite and its metabolites, it is of high interest to study mechanisms underlying the low NO_2_ level maintenance in the cell. During anaerobic growth of *Escherichia coli* the main nitrite-reducing enzymes are NrfA and NirB nitrite reductases. NrfA reductase is localized in the cell periplasm and uses NO_2_ as an electron acceptor to create a proton gradient; NirB reductase is restricted to the cytoplasm and metabolizes excessive nitrite inside the cell, the uptake of which is mediated by the transporter protein NirC. While it is known that these three systems, periplasmic, cytoplasmic and transport, determine nitrite uptake and assimilation in the cell as well as its excretion, little is known about their co-ordination.

**Results:**

Using a mathematical model describing the nitrite utilization in *E. coli* cells cultured in a flow chemostat, the role of enzymes involved in nitrite metabolism and transport in controlling nitrite intracellular levels was investigated. It was demonstrated that the model adapted to the experimental data on expression *of nrfA* and *nirB* genes encoding NrfA and NirB nitrite reductases, can describe nitrite accumulation kinetics in the chemostat in the millimolar range of added substrate concentrations without any additional assumptions. According to the model, in this range, low intracellular nitrite level, weakly dependent on its concentration in the growth media, is maintained (mcM). It is not sufficient to consider molecular-genetic mechanisms of NrfA reductase activity regulation to describe the nitrite accumulation dynamics in the chemostat in the micromolar range (≤1 mM) of added nitrite concentrations. Analysis of different hypotheses has shown that the mechanism of local enzyme concentration change due to membrane potential-induced diffusion from the cytoplasm to the periplasm at low nitrite levels is sufficient to explain the nitrite accumulation dynamics in the chemostat.

**Conclusions:**

At nitrite concentrations in the media more than 2 mM, the model adapted to the experimental data on nitrite utilization dynamics in *E. coli* cells cultured in the flow chemostat demonstrates the largest contribution of genetic mechanisms involved in *nrf* and *nir* operons activity regulation to the control of nitrite intracellular levels. The model predicts a significant contribution of the membrane potential to the periplasmic NrfA nitrite reductase activity regulation and nitrite utilization dynamics at substrate concentrations ≤1 mM.

## Background

During anaerobic growth *E. coli* uses different electron acceptors in the electron transport chain, including nitrite (NO_2_). Given the high toxicity of nitrite, expression regulation of genes involved in the nitrite-associated electron transport chain is closely linked to the expression regulation of nitrite metabolism genes. Nitrite reductases NrfA (EC 1.7.2.2) and NirB (EC 1.7.1.4) are two main components of the nitrite-reducing system. These enzymes have different metabolite activities and localizations in the cell. NrfA reductase is a respiratory enzyme localized in the cell periplasm; it takes part in the proton gradient generation and uses NO_2_ as an electron acceptor. NirB reductase is restricted to the cytoplasm and, most probably, has excessive nitrite detoxification as its main role in the cell. Both enzymes catalyze the reduction of NO_2_ to ammonium, but NrfA is most active at low nitrite levels, whereas NirB activity is observed only at high nitrite levels [1]. Differential expression of *nrf* and *nir* operons, encoding NrfA and NirB reductases, respectively, enables such a combination of NrfA and NirB reductase activities. The *nrf* and *nir* operons expression is controlled by transcription factors, NarL and NarP, the activity of which depends on NarQ and NarX kinases [2].

Unlike *nir* operon, the expression of which is always positively regulated by nitrite [3], the *nrf* operon has regulatory region that allows its expression activation at low nitrite levels and expression inhibition at high nitrite levels [1, 4]. As a result, at nitrite concentrations in the media > 2 mM, genetic system that encodes NrfA reductase and allows nitrite metabolism in the periplasm of the cell switches to a more effective in terms of nitrite recycling genetic system, that encodes NirC transporter and NirB nitrite reductase and allows nitrite transport into the cell and its further recycle by the cytoplasmic reductase [1].

Due to a high toxicity of nitrite, cell transport system plays a crucial role in the nitrite level regulation in the cell. Three transport proteins involved in the nitrite transmembrane passage are known: NarK, NarU and NirC [5, 6], but the nitrite transport protein NirC is known to have the highest activity among three [6]. Localization of *nirС* gene, encoding the NirC protein, in the same operon as *nirB* gene demonstrates a tight connection between intracellular nitrite utilization and nitrite import/export systems at high nitrite levels; however, complex interplay between periplasmic and cytoplasmic systems, which determines the nitrite accumulation and utilization in the cell as well as its export from the cell, represents a significant knowledge gap.

The existing amount of qualitative and quantitative data on *nrfA, nirB* and *nirС* genes expression regulation and nitrite accumulation dynamics during *E. coli* stationary phase growth in chemostat [1, 7], allowed us to develop a mathematical model of nitrite intracellular utilization and investigate how NirB and NrfA reductases and NirC transporter contribute to this process. Using this model, mechanisms involved in regulation of these proteins’ activities were investigated for their roles in controlling the intracellular nitrite levels.

## Mathematical model of nitrite utilization in *E. coli* cells

**Fig. 1:**
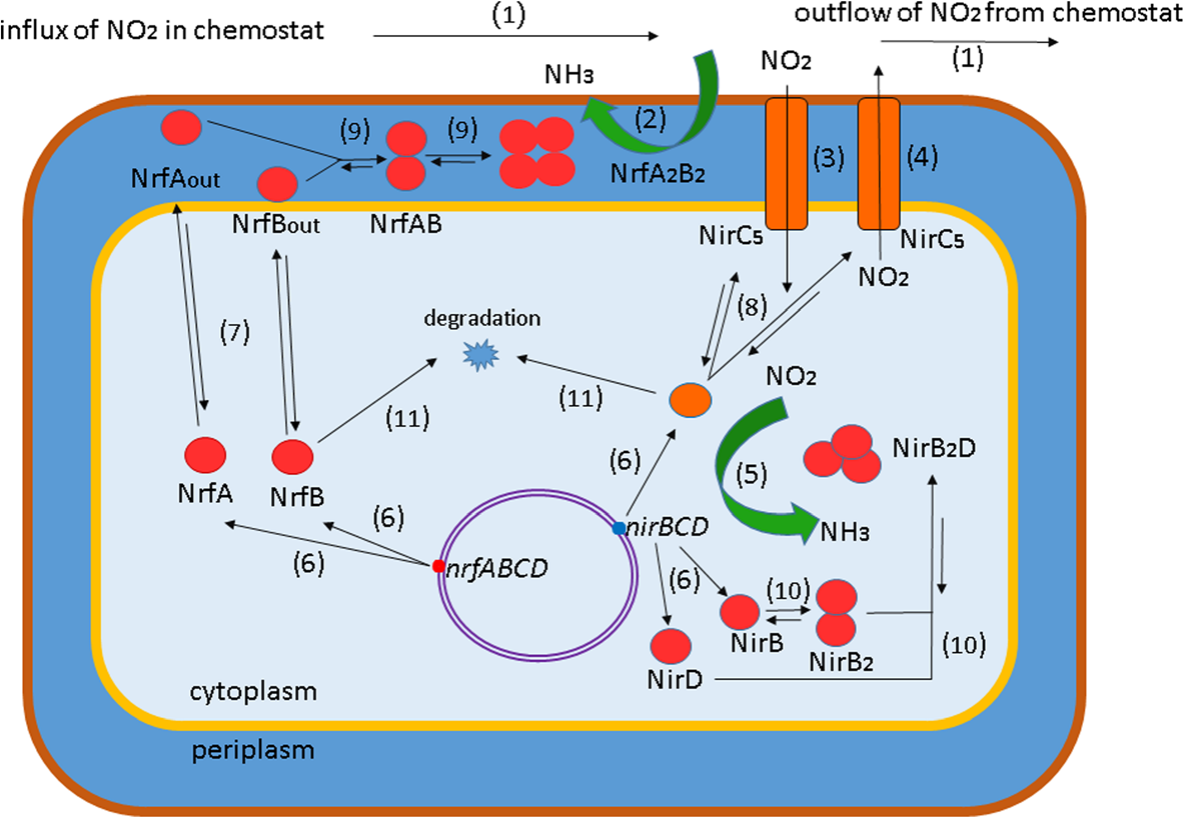
Scheme of the processes occurring in the chemostat during nitrite utilization in *E. coli* cells. (1) – nitrite inflow and outflow at steady-state flow rate; (2) – reduction of extracellular nitrite to ammonium mediated by periplasmic NrfA reductase; (3),(4) – import of the extracellular nitrite into the cell and its export from the cell into the chemostat mediated by the NirC transporter; (5) – intracellular nitrite utilization mediated by cytoplasmic NirB reductase; (6) – NrfA and NrfB proteins synthesis from the *nrf* operon mRNA and NirB, NirC and NirD synthesis from the *nir* operon mRNA; (7) – transport of the Nrf reductase A and B subunits from the cytoplasm to the periplasm and back; (8) – formation of the NirC transporter active pentameric form; (9) – formation of the NirB reductase active heteromeric form; (10) – formation of the Nrf active heterotetrameric form; (11) – degradation of proteins and their complexes (degradation process is illustrated in the figure only for two monomers)

The exact conditions of *E. coli* cells cultivation in a nitrite-supplied flow chemostat are reproduced in the model. The model is adapted to the experimental data on nitrite accumulation dynamics in a chemostat and on the inducer levels dependent *nrf* and *nir* operons expression [1, 7]. A steady-state growth rate and cell culture density in the chemostat was achieved by fixating the glucose uptake [7]. The model of nitrite utilization in *E. coli* cells cultured in a chemostat consists of several elementary subsystems. Subsystems are detailed in accordance with the process scheme depicted in Fig. 1 and are described below. When describing these processes, generalized Hill functions were applied [8].

### Elementary subsystems of the model

#### Subsystem (1) describes the nitrite flow through the chemostat

Nitrite inflow rate into the chemostat is a constant parameter throughout the experiment and nitrite outflow rate from the chemostat is proportional to its current concentration, so the total nitrite flow rate through the chemostat can be described with the equation

$$ \frac{du}{dt}={V}_{in}- kflow\cdot u, $$

where *u* – extracellular nitrite concentration in the chemostat, *V*_*in*_ (mM/s) – nitrite inflow rate into the chemostat, *kflow* (sec^−1^) – the rate constant for the nitrite outflow. Later on, for convenience, *V*_*in*_ parameter will be presented as *V*_*in*_ = *kflow* ⋅ *s*, where *s* is a nitrite concentration established in the chemostat at a given *V*_*in*_ and *kflow* values in the absence of the cell culture. Later on we will name this parameter «added nitrite».

#### Subsystem (2) describes the reduction of extracellular nitrite to ammonium mediated by the periplasmic formate-dependent NrfA reductase

The reduction of extracellular nitrite to ammonium by periplasmic formate-dependent NrfA reductase is described in accordance with the stoichiometric scheme: NO_2_^−^ + 6е^−^ + 7H^+^ → NH_3_ + 2H_2_O with the simplest Michaelis–Menten model [9–11]

$$ \left\{\begin{array}{l}\frac{du}{dt}=-C{k}_{dis,Nrf{A}_2{B}_2u}\left(u\times Nrf{A}_2{B}_2/{K}_{M,Nrf}-Nrf{A}_2{B}_2u\right),\\ {}\frac{dNrf{A}_2{B}_2}{dt}={k}_{dis,Nrf{A}_2{B}_2u}\left(u\times Nrf{A}_2{B}_2/{K}_{M,Nrf}-Nrf{A}_2{B}_2u\right)-{k}_{cat,Nrf}Nrf{A}_2{B}_2u,\\ {}\frac{dNrf{A}_2{B}_2}{dt}=-{k}_{dis,Nrf{A}_2{B}_2u}\left(u\times Nrf{A}_2{B}_2/{K}_{M,Nrf}-Nrf{A}_2{B}_2u\right)+{k}_{cat,Nrf}Nrf{A}_2{B}_2u,\end{array}\right. $$

where *NrfA*_2_*B*_2_(*u*) – nitrite-dependent NrfA reductase concentration in the cell periplasm (deduced in the subsystem (7)), *C* – cell volume fraction in the chemostat volume, *k*_*cat*,*Nrf*_ – catalytic constant of NrfA reductase turnover, *K*_*M*,*Nrf*_ – the Michaelis constant.

#### Subsystem (3) describes the extracellular nitrite import into the cell

It is known that extracellular nitrite transport from the environment into the cell is mediated by the NirC, NarU и NarK transporter proteins [5, 6], but due to significantly higher NirC nirtrite transport activity, compared with NarU and NarK [6], only NirC transporter function was considered in the model.

The structure of the *E. coli* NirC transporter catalytically active form is not known. We assume that it should be similar to the *Salmonella typhimurium* NirC structure because both proteins belong to one family [12]; that is why in the model NirC is assumed to be a pentameric protein. The mechanism of nitrite import by the NirC transporter is also not known, therefore we described this process rate with the simplest system, resulted from the simplest Michaelis–Menten model

$$ \left\{\begin{array}{l}\frac{du}{dt}=-C{k}_{dis,Nir{C}_5u}\left(u\times Nir{C}_5/{K}_{M, NirCin}-Nir{C}_5u\right),\\ {}\frac{dNir{C}_5}{dt}={k}_{dis,Nir{C}_5u}\left(u\times Nir{C}_5/{K}_{M, NirCin}-Nir{C}_5u\right)-{k}_{cat, NirCin}Nir{C}_5u,\\ {}\frac{dNir{C}_5u}{dt}=-{k}_{dis,Nir{C}_5u}\left(u\times Nir{C}_5/{K}_{M, NirCin}-Nir{C}_5u\right)+{k}_{cat, NirCin}Nir{C}_5u,\end{array}\right. $$

where *NirC*_5_ – nitrite-dependent concentration of the NirC transporter active form in the cell periplasm, ${k}_{dis,Nir{C}_5u}$ – the rate constant for the *u* × *NirC*_5_ complex dissociation into subunits, *k*_*cat*,*NirCin*_ – the rate constant for the nitrite import by the NirC transporter, *K*_*M*,*NirCin*_ – the Michaelis constant.

#### Subsystem (4) describes intracellular nitrite export from the cell to the chemostat

In the model intracellular nitrite export from the cell to the chemostat, as well as its import, is mediated only by the NirC transporter. The NarU and NarK transporters contribution to this process was not considered [6]. Because the mechanism of nitrite import by the NirC transporter is not yet studied, the rate of this process is described with the simplest equation

$$ \left\{\begin{array}{l}\frac{dw}{dt}=-{k}_{dis,Nir{C}_5w}\left(w\times Nir{C}_5/{K}_{M, NirCout}-Nir{C}_5w\right),\\ {}\frac{dNir{C}_5}{dt}={k}_{dis,Nir{C}_5w}\left(w\times Nir{C}_5/{K}_{M, NirCout}-Nir{C}_5w\right)-{k}_{cat, NirCout}Nir{C}_5w,\\ {}\frac{dNir{C}_5w}{dt}=-{k}_{dis,Nir{C}_5w}\left(w\times Nir{C}_5/{K}_{M, NirCout}-Nir{C}_5w\right)+{k}_{cat, NirCout}Nir{C}_5w,\end{array}\right. $$

where *w* –intracellular nitrite concentration, ${k}_{dis,Nir{C}_5w}$ – the rate constant for the *w* × *NirC*_5_ complex dissociation into subunits, *k*_*cat*,*NirCout*_ – the rate constant for the nitrite export by the NirC transporter, *K*_*M*,*NirCout*_ – the Michaelis constant.

#### Subsystem (5) describes the intracellular nitrite utilization mediated by the NADN-dependent NirB reductase

In the model this process is described in accordance with the following stoichiometric scheme:

$$ {{\mathrm{NO}}_2}^{-} + 3\mathrm{NADH} + 5{\mathrm{H}}^{+}\ \to\ {{\mathrm{NH}}_4}^{+} + 3{\mathrm{NAD}}^{+} + 2{\mathrm{H}}_2\mathrm{O}. $$

The utilization rate is calculated with the Michaelis–Menten law [13, 14]

$$ \left\{\begin{array}{l}\frac{dw}{dt}=-{k}_{dis,Nir{B}_2Dw}\left(w\times Nir{B}_2D/{K}_{M, NirB}-Nir{B}_2Dw\right),\\ {}\frac{dNir{B}_2D}{dt}={k}_{dis,Nir{B}_2Dw}\left(w\times Nir{B}_2D/{K}_{M, NirB}-Nir{B}_2Dw\right)-{k}_{cat, NirB}Nir{B}_2Dw,\\ {}\frac{dNir{B}_2Dw}{dt}=-{k}_{dis,Nir{C}_5w}\left(w\times Nir{C}_5/{K}_{M, NirCout}-Nir{C}_5w\right)+{k}_{cat, NirB}Nir{B}_2Dw,\end{array}\right. $$

where *NirB*_2_*D* – nitrite-dependent concentration of the NirB reductase active form, ${k}_{dis,Nir{B}_2Dw}$ – the rate constant for the *w* × *NirB*_2_*D* molecular complex dissociation into subunits, *k*_*cat*,*NirB*_ – the recycle catalytic constant, *K*_*M*,*NirB*_ – the Michaelis constant.

#### Subsystem (6) describes NrfA, NrfB, NirB, NirC и NirD proteins synthesis

**Fig. 2:**
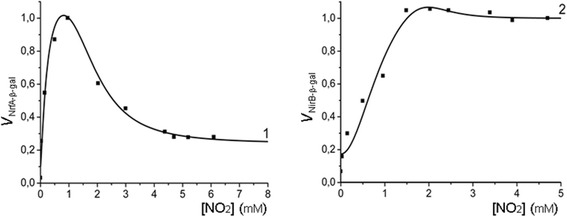
Effect of nitrite concentration on *nrfABCD* and *nirBDC* operons expression during anaerobic cell growth. Dots represent experimental values of activities of the NrfA-β-gal and NirB-β-gal chimeric proteins measured in [1] and approximating their theoretical curves *m*_*Nrf*_ (*u*) (curve 1) and *m*_*Nir*_(*u*) (curve 2). The X-axis – steady-state nitrite concentration (*u*) in the chemostat (mM); the Y-axis (left) – the NrfA-β-gal chimeric protein relative activity, (right) – the NrfB-β-gal chimeric protein relative activity. The steady-state nitrite concentration in the chemostat used as a scale bar on the X-axis was calculated earlier [35], and based on the data from the previous research [1]

In *E. coli* cells NrfA and NrfB proteins are synthesized from mRNA templates of the *nrf* operon and NirB, NirC and NirD proteins – from mRNA templates of the *nir* operon. The *nrf* operon mRNA concentration in the cell that exists in the chemostat at a current nitrite concentration *u* is assumed to be proportional to the relative NrfA-β-gal chimeric protein activity observed at the same nitrite concentration (Fig. 2, curve 1); and the *nir* operon mRNA concentration in the same cell is assumed to be proportional to the relative NirB-β-gal chimeric protein activity (Fig. 2, curve 2), measured by Wang and co-authors [1].

Activities of NrfA-β-gal and NirB-β-gal chimeric proteins are dependent on the nitrite concentration and were approximated with generalized Hill functions,

$$ {m}_{Nrf}(u)=\frac{1+{\delta}_{nrf,1}\times {\left(\frac{u}{K_{nrf,1}}\right)}^{h_{nrf,1}}}{1+{\left(\frac{u}{K_{nrf,1}}\right)}^{h_{nrf,1}}}\times \frac{1+{\delta}_{nrf,2}\times {\left(\frac{u}{K_{nrf,2}}\right)}^{h_{nrf,2}}}{1+{\left(\frac{u}{K_{nrf,2}}\right)}^{h_{nrf,2}}} $$

$$ {m}_{Nir}(u)=\frac{1+{\delta}_{nir,1}\times {\left(\frac{u}{K_{nir,1}}\right)}^{h_{nir,1}}+{\delta}_{nir,2}\times {\left(\frac{u}{K_{nir,2}}\right)}^{h_{nir,2}}}{1+{\left(\frac{u}{K_{nir,1}}\right)}^{h_{nir,1}}+{\left(\frac{u}{K_{nir,2}}\right)}^{h_{nir,2}}}. $$

Optimal parameters of the *m*_*Nrf*_ (*u*) and *m*_*Nir*_ (*u*) functions were estimated with the gradient descent algorithm and are represented in the Appendix: Table 1.

As a result, synthesis of NrfA and NrfB proteins can be described with the following system of differential equations:

$$ \left\{\begin{array}{l}\frac{dNrf{A}_c}{dt}=k{s}_{Nrf,{A}_c}\cdot {m}_{Nrf}(u),\\ {}\frac{dNrf{B}_c}{dt}=k{s}_{Nrf,{B}_c}\cdot {m}_{Nrf}(u),\end{array}\right. $$

where *NrfA*_*c*_ and *NrfB*_*c*_ – concentrations of NrfA and NrfB proteins in the cytoplasm, *ks*_*Nrf*_ – the rate constant for the synthesis of NrfA and NrfB proteins, and synthesis of NirB, NirC and NirD proteins can be described with the following system of equations:

$$ \left\{\begin{array}{l}\frac{dNirB}{dt}=k{s}_{Nir,B}\cdot {m}_{Nir}(u),\\ {}\frac{dNirC}{dt}=k{s}_{Nir,C}\cdot {m}_{Nir}(u),\\ {}\frac{dNirD}{dt}=k{s}_{Nir,D}\cdot {m}_{Nir}(u),\end{array}\right. $$

where *NirB*, *NirC*, *NirD* – concentrations of NirB, NirC and NirD proteins in the cytoplasm, *ks*_*Nir*_ – the rate constant for the protein synthesis.

It is considered that the NirC protein translation level is more than five times higher than such of the NirB protein in the millimolar range of nitrite concentration [15]

#### Subsystem (7) describes transport of the NrfA and NrfB subunits of the Nrf nitrite reductase from the cytoplasm to the periplasm

The Nrf enzyme is assumed to be transported from the cytoplasm to the periplasm as monomer subunits NrfA and NrfB, which are synthezised in the cytoplasm.

We also assume that the specific rate constant for the Nrf reductase A and B monomers transport from the cytoplasm, where their synthesis takes place, to the periplasmic space, where the active enzyme is being formed, is a variable that depends on the membrane potential: *kt*_*Nrf*_ ⋅ *k*_*U*_(*U*(*s*)). Generalized Hill functions is used for the *k*_*U*_ description

$$ k{t}_{Nrf,cp,U}\left(U(s)\right)=k{t}_{Nrf,cp}\left(1+{d}_UU(s)\right), $$

whose value is made dependent on the *U* function, named the nominal potential.

In its turn, the *U* function mimics the membrane potential that changes in relation to the nitrite concentration in the media. Due to the lack of knowledge on the mechanism of *E. coli* natural membrane potential formation, the phenomenological function *U*, which qualitatively reproduces the experimental data received by Motteram et al. [16], was used in the model. According to these data, the membrane potential highest value was observed in the 0.1-1 mM nitrite concentration range and was stable starting from the 0.2 mM nitrite concentrations, and decreased outside this range. To reproduce this phenomenon, we used a modified formula (11) from the previous research [17]:

$$ U(s)=\frac{{\left(\frac{s}{K_{pmf,1}}\right)}^{h_{pmf,1}}+\delta \times {\left(\frac{s}{K_{pmf,2}}\right)}^{h_{pmf,2}}+{\omega}_2\times {\left(\frac{s}{K_{pmf,3}}\right)}^{h_{pmf,3}}}{1+{\left(\frac{s}{K_{pmf,1}}\right)}^{h_{pmf,1}}+{\left(\frac{s}{K_{pmf,2}}\right)}^{h_{pmf,2}}+{\omega}_1\times {\left(\frac{s}{K_{pmf,3}}\right)}^{h_{pmf,3}}}. $$

**Fig. 3:**
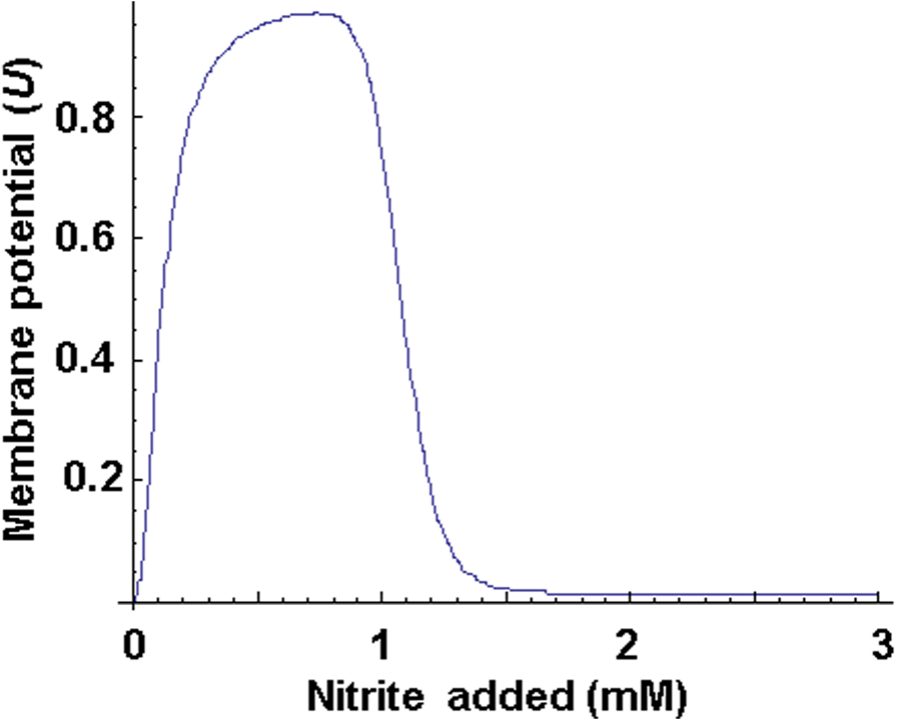
The nominal membrane potential *U*(*s*) in relation to the added nitrite level. The membrane potential value is presented in arbitrary units

Further, we estimated parameter values for the given function (values are represented in the Appendix: Table 1) so that its behavior was qualitatively consistent with the above listed features. Qualitative behavior of the *U*(*s*) function is presented in the Fig. 3.

The system of differential equations describing the transport process is as follows:

$$ \left\{\begin{array}{l}\frac{dNrf{A}_c}{dt}=-\left(k{t}_{Nrf,cp,U}\left(U(s)\right)\cdot Nrf{A}_c-k{t}_{Nrf,pc}\cdot NrfA\right),\\ {}\frac{dNrf{B}_c}{dt}=-\left(k{t}_{Nrf,cp,U}\left(U(s)\right)\cdot Nrf{B}_c-k{t}_{Nrf,pc}\cdot NrfB\right),\\ {}\frac{dNrf A}{dt}={\delta}_{peripl}\left(k{t}_{Nrf,cp,U}\left(U(s)\right)\cdot Nrf{A}_c-k{t}_{Nrf,pc}\cdot NrfA\right),\\ {}\frac{dNrf B}{dt}={\delta}_{peripl}\left(k{t}_{Nrf,cp,U}\left(U(s)\right)\cdot Nrf{B}_c-k{t}_{Nrf,pc}\cdot NrfB\right),\end{array}\right. $$

where *NrfA*_*c*_ and *NrfB*_*c*_ – concentrations of NrfА and NrfВ proteins in the cytoplasm, *NrfA* and *NrfB* – concentrations of NrfА and NrfВ proteins in the periplasm, *kt*_*Nrf*,*c* *p*_ – the rate constant for the NrfА and NrfВ proteins transport from the cytoplasm to the periplasm, *kt*_*Nrf*,*p* *c*_ – the rate constant for the NrfА and NrfВ proteins transport from the periplasm to the cytoplasm, *δ*_*peripl*_ – ratio of cytoplasmic volume to periplasmic volume.

#### Subsystem (8) describes the NirC transporter active form formation

As stated above, the structure of the *E. coli* NirC transporter catalytically active form is not known. Because both *E. coli* NirC and *S. typhimurium* NirC proteins belong to the same family, pentameric NirC protein of *S. typhimurium* was used for modeling the *E. coli* NirC [12]. Accordingly, the complex formation is described based on a fifth-order reaction

$$ NirC+ NirC+ NirC+ NirC+ NirC\underset{k_{dis,Nir{C}_5}}{\overset{k_{form,Nir{C}_5}}{\rightleftarrows }}Nir{C}_5 $$

which implies the following system of differential equations

$$ \left\{\begin{array}{l}\frac{dNir C}{dt}=5{k}_{dis,{C}_5}\left(Nir{C}_5-{\left({K}_{dis,{C}_5}\right)}^{-4}Nir{C}_5\right),\\ {}\frac{dNir{C}_5}{dt}=-{k}_{dis,{C}_5}\left(Nir{C}_5-{\left({K}_{dis,{C}_5}\right)}^{-4}Nir{C}_5\right),\end{array}\right. $$

where *NirC* and *NirC*_5_ – concentrations of NirC monomeric protein and NirC_5_ pentameric protein, ${k}_{form,{C}_5},{k}_{dic,{C}_5}$ – the rate constants for the forward and reverse reactions, ${K}_{dic,{C}_5}=\sqrt[4]{\frac{k_{dic,{C}_5}}{k_{form,{C}_5}}}$ – the dissociation constant of the NirC_5_ complex.

#### Subsystem (9) describes the periplasmic Nrf reductase active form formation

It is assumed in the model that under physiological conditions NrfA operates as part of the heterotetrameric NrfA_2_–NrfB_2_ complex [18, 19]. Genes encoding NrfA and NrfB subunits of the enzyme are clustered in one operon and are translated in the *E. coli* cell in equimolar amounts [20]. The Nrf active form (*NrfA*_2_*В*_2_) concentration in the periplasm was calculated with the biochemical model [18, 19]:

$$ NrfA+ NrfB\underset{k_{dis, AB}}{\overset{k_{form, AB}}{\rightleftarrows }} NrfAB,\kern0.24em  NrfAB+ NrfAB\underset{k_{dis,{A}_2{B}_2}}{\overset{k_{form,{A}_2{B}_2}}{\rightleftarrows }}Nrf{A}_2{B}_2 $$

This implies a system of differential equations that describes the NrfAB and NrfA_2_B_2_ complexes formation and degradation in the periplasm, as well as NrfA and NrfB proteins degradation in the periplasm:

$$ \left\{\begin{array}{l}\frac{dNrf A}{dt}={k}_{dis, AB}\left( NrfAB-{K}_{dis, AB}^{-1} NrfA\cdot NrfB\right),\\ {}\frac{dNrf B}{dt}={k}_{dis, AB}\left( NrfAB-{K}_{dis, AB}^{-1} NrfA\cdot NrfB\right),\\ {}\frac{dNrf A B}{dt}=-{k}_{dis, AB}\left( NrfAB-{K}_{dis, AB}^{-1} NrfA\cdot NrfB\right)+2{k}_{dis,{A}_2{B}_2}\left(Nrf{A}_2{B}_2-{K}_{dis,{A}_2{B}_2}^{-1} NrfA{B}^2\right),\\ {}\frac{dNrf{A}_2{B}_2}{dt}=-{k}_{dis,{A}_2{B}_2}\left(Nrf{A}_2{B}_2-{K}_{dis,{A}_2{B}_2}^{-1} NrfA{B}^2\right),\end{array}\right. $$

where *NrfA, NrfB* – concentrations of free NrfA and NrfB monomeric proteins in the periplasm, *NrfAB –*NrfAB complex concentration in the periplasm, *NrfA*_2_*B*_2_ – concentration of the active heterotetrameric form (NrfA_2_B_2_) of the Nrf enzyme, *k*_*form*,*AB*_, ${k}_{form,{A}_2{B}_2}$ and *k*_*dis*,*AB*_, ${k}_{dis,{A}_2{B}_2}$*–* the rate constants for the forward and reverse reactions, ${K}_{dis, AB}=\frac{k_{dis, AB}}{k_{form, AB}}$, ${K}_{dis,{A}_2{B}_2}=\frac{k_{dis,{A}_2{B}_2}}{k_{form,{A}_2{B}_2}}$*–* the dissociation constants for the NrfAB and NrfA_2_B_2_ complexes.

#### Subsystem (10) describes the active form formation of the cytoplasmic NirB reductase

According to [15], it is assumed in the model that under physiological conditions NirB nitrite reductase operates as part of NirB_2_NirD the heterotrimeric complex. The concentration of the NirB reductase active form was evaluated with the biochemical model:

$$ NirB+ NirB\underset{k_{dis,{B}_2}}{\overset{k_{form,{B}_2}}{\rightleftarrows }}Nir{B}_2,\kern0.24em Nir{B}_2+ NirD\underset{k_{dis,{B}_2D}}{\overset{k_{form,{B}_2D}}{\rightleftarrows }}Nir{B}_2D $$

On this basis, a system of differential equations describing the NirB_2_ and NirB_2_NirD complexes formation and degradation as well as NirB and NirD proteins degradation in the cytoplasm is as follows:

$$ \left\{\begin{array}{l}\frac{dNir B}{dt}=2{k}_{dis,{B}_2}\left(Nir{B}_2-{K}_{dis,{B}_2}^{-1}Nir{B}^2\right),\\ {}\frac{dNir{B}_2}{dt}=-{k}_{dis,{B}_2}\left(Nir{B}_2-{K}_{dis,{B}_2}^{-1}Nir{B}^2\right)+{k}_{dis,{B}_2D}\left(Nir{B}_2D-{K}_{dis,{B}_2D}^{-1}Nir{B}_2\times NirD\right),\\ {}\frac{dNir D}{dt}={k}_{dis,{B}_2D}\left(Nir{B}_2D-{K}_{dis,{B}_2D}^{-1}Nir{B}_2\times NirD\right),\\ {}\frac{dNir{B}_2D}{dt}=-{k}_{dis,{B}_2D}\left(Nir{B}_2D-{K}_{dis,{B}_2D}^{-1}Nir{B}_2\times NirD\right),\end{array}\right. $$

where *NirB*, *NirD* –intracellular concentrations of the NirB and NirD monomeic proteins*, NirB*_2_ – intracellular concentration of the NirB_2_ dimer, *NirB*_2_*D* – intracellular concentration of the cytoplasmic nitrite reductase active form, which is a NirB_2_D trimer, $$ {k}_{dis,{B}_2},{k}_{form,{B}_2}, $$$$ {k}_{dis,{B}_2},{k}_{form,{B}_2} $$*–* the rate constants for the forward and reverse reactions, $$ {K}_{dis,{B}_2}=\frac{k_{dis,{B}_2}}{k_{form,{B}_2}} $$, $$ {K}_{dis,{B}_2D}=\frac{k_{dis,{B}_2D}}{k_{form,{B}_2D}} $$*–* the dissociation constants for the NirB_2_ and NirB_2_NirD complexes.

#### Subsystem (11) describes the degradation of proteins and their complexes as well as their dilution during cell growth

Degradation processes of proteins and their complexes are described by the linear monomolecular reaction. The dilution rate of the substance intracellular concentration due to the cell growth, when concentration is *x*, in general equals (*V*’/*V*)*x*, where *V* – current cell volume and *V’* – cell volume growth rate. Because the growth rate in the chemostat is synchronized with the outflow rate, the average cell growth rate constant is equal to the outflow rate constant [7]; as a result, we have the following system

$$ \frac{dX}{dt}=\left({k}_{d,X}+ kflow\right)X,X=w,{NrfA}_c{Nrf}_c, NrfA, NrfB, NirC, NirB, NirD, NrfA B,{NrfA}_2{B}_2,{NirB}_2,{NirB}_2D,{NirC}_{5.} $$

#### Disequilibrium model assemblage

To assemble a disequilibrium model from the equations describing (1)–(11) subsystems, the rate law was used, according to which the total change rate of a particular substance concentration equals to the change rates sum of a substance concentrations in each local process. In this case, the local processes are the (1)–(11) subsystems, and the corresponding differential equations predetermine the local rates.

As a result, we have a system of differential equations (1), which is listed below.

1 $$ \left\{\begin{array}{l}\frac{du}{dt}={k}_{flow}\left(s-u\right)+C\cdot \left[\begin{array}{l}{k}_{dis,Nrf{A}_2{B}_2u}\left(Nrf{A}_2{B}_2u-{K}_{dis,Nrf{A}_2{B}_2u}^{-1}Nrf{A}_2{B}_2\times u\right)+\\ {}+{k}_{dis,Nir{C}_5}\left(Nir{C}_5-{K}_{dis,Nir{C}_5u}^{-1}Nir{C}_5\times u\right)+{k}_{cat, NirC out}Nir{C}_5w\end{array}\right],\\ {}\frac{dw}{dt}={k}_{cat, NirC in}Nir{C}_5u+{k}_{dis,Nir{C}_5w}\left(Nir{C}_5w-{K}_{dis,Nir{C}_5w}^{-1}Nir{C}_5\times w\right)+\\ {}\kern5.64em +{k}_{dis,Nir{B}_2Dw}\left(Nir{B}_2Dw-{K}_{dis,Nir{B}_2Dw}^{-1}Nir{B}_2D\times w\right)- kflow\cdot w,\\ {}\frac{dNir C}{dt}=k{s}_{Nir}{m}_{Nir}(u)+5{k}_{dis,Nir{C}_5}\left(Nir{C}_5-{K}_{dis,Nir{C}_5}^{-4}Nir{C}^5\right)-\left({k}_{d, NirC}+ kflow\right) NirC,\\ {}\frac{dNir{C}_5}{dt}={k}_{cat, NirC in}Nir{C}_5u+{k}_{cat, NirC out}Nir{C}_5w-{k}_{dis,Nir{C}_5}\left(Nir{C}_5-{K}_{dis,Nir{C}_5}^{-4}Nir{C}^5\right)-\\ {}-{k}_{dis,Nir{C}_5u}\left(Nir{C}_5u-{K}_{dis,Nir{C}_5u}^{-1}Nir{C}_5\times u\right)-{k}_{dis,Nir{C}_5w}\left(Nir{C}_5w-{K}_{dis,Nir{C}_5w}^{-1}Nir{C}_5\times w\right)-\left({k}_{d,Nir{C}_5}+ kflow\right)Nir{C}_5,\\ {}\frac{dNir{C}_5u}{dt}=-{k}_{dis,Nir{C}_5w}\left(Nir{C}_5u-{K}_{dis,Nir{C}_5u}^{-1}Nir{C}_5\times u\right)-\left({k}_{cat, NirC in}+ kflow\right)Nir{C}_5u,\\ {}\frac{dNir{C}_5w}{dt}=-{k}_{dis,Nir{C}_5w}\left(Nir{C}_5w-{K}_{dis,Nir{C}_5w}^{-1}Nir{C}_5\times w\right)-\left({k}_{cat, NirC out}+ kflow\right)Nir{C}_5w,\\ {}\frac{dNir B}{dt}=k{s}_{Nir}{m}_{Nir}(u)+2{k}_{dis,Nir{B}_2}\left(Nir{B}_2-{K}_{dis,Nir{B}_2}^{-1}Nir{B}^2\right)-\left({k}_{d, NirB}+ kflow\right) NirB,\\ {}\frac{dNir D}{dt}=k{s}_{Nir}{m}_{Nir}(u)+{k}_{dis,Nir{B}_2D}\left(Nir{B}_2D-{K}_{dis,Nir{B}_2D}^{-1}Nir{B}_2\times NirD\right)-\left({k}_{d, NirD}+ kflow\right) NirD,\\ {}\frac{dNir{B}_2}{dt}={k}_{dis,Nir{B}_2}\left(Nir{B}_2-{K}_{dis,Nir{B}_2}^{-1}Nir{B}^2\right)+\\ {}\kern5.4em +{k}_{dis,Nir{B}_2D}\left(Nir{B}_2D-{K}_{dis,Nir{B}_2D}^{-1}Nir{B}_2\times NirD\right)-\left({k}_{d,Nir{B}_2}+ kflow\right)Nir{B}_2,\\ {}\frac{dNir{B}_2D}{dt}=-{k}_{dis,Nir{B}_2D}\left(Nir{B}_2D-{K}_{dis,Nir{B}_2D}^{-1}Nir{B}_2\times NirD\right)+{k}_{cat, NirB}Nir{B}_2Dw+\\ {}\kern3.24em +{k}_{dis,Nir{B}_2Dw}\left(Nir{B}_2Dw-{K}_{dis,Nir{B}_2Dw}^{-1}Nir{B}_2D\times w\right)-\left({k}_{d,Nir{B}_2D}+ kflow\right)Nir{B}_2D,\\ {}\frac{dNir{B}_2Dw}{dt}=-{k}_{dis,Nir{B}_2Dw}\left(Nir{B}_2Dw-{K}_{dis,Nir{B}_2Dw}^{-1}Nir{B}_2D\times w\right)-\left({k}_{cat, NirB}+ kflow\right)Nir{B}_2Dw,\\ {}\frac{dNrf{A}_c}{dt}=k{s}_{Nrf}{m}_{Nrf}(u)-\left(k{t}_{Nrf,cp,U}\left(U(s)\right)Nrf{A}_c-k{t}_{Nrf,pc} NrfA\right)-\left({k}_{d,Nrf{A}_c}+ kflow\right)Nrf{A}_c,\\ {}\frac{dNrf{B}_c}{dt}=k{s}_{Nrf}{m}_{Nrf}(u)-\left(k{t}_{Nrf,cp,U}\left(U(s)\right)Nrf{B}_c-k{t}_{in,pc} NrfB\right)-\left({k}_{d,Nrf{B}_c}+ kflow\right)Nrf{B}_c,\\ {}\frac{dNrf A}{dt}={\delta}_{peripl}\cdot \left(k{t}_{Nrf,cp,U}\left(U(s)\right)Nrf{A}_c-k{t}_{Nrf,pc} NrfA\right)\\ {}\kern6.96em +{k}_{form, NrfA B}\left({K}_{dis, NrfA B} NrfA B- NrfA\cdot NrfB\right)-\left({k}_{d, NrfA}+ kflow\right) NrfA,\\ {}\frac{dNrf B}{dt}={\delta}_{peripl}\cdot \left(k{t}_{Nrf,cp,U}\left(U(s)\right)Nrf{B}_c-k{t}_{Nrf,pc} NrfB\right)\\ {}\kern6.96em +{k}_{form, NrfA B}\left({K}_{dis, NrfA B} NrfA B- NrfA\cdot NrfB\right)-\left({k}_{d, NrfB}+ kflow\right) NrfB,\\ {}\frac{dNrf A B}{dt}=2{k}_{dis,Nrf{A}_2{B}_2}\left(Nrf{A}_2{B}_2-{K}_{dis,Nrf{A}_2{B}_2}^{-1}{(NrfAB)}^2\right)\\ {}\kern6.72em -{k}_{dis, NrfA B}\left( NrfAB-{K}_{dis, NrfA B}^{-1} NrfA\cdot NrfB\right)-\left({k}_{d, NrfA B}+ kflow\right) NrfA B,\\ {}\frac{dNrf{A}_2{B}_2}{dt}={k}_{dis,Nrf{A}_2{B}_2u}\left(Nrf{A}_2{B}_2u-{K}_{dis,Nrf{A}_2{B}_2u}^{-1}Nrf{A}_2{B}_2\times u\right)+{k}_{cat,Nrf{A}_2{B}_2u}Nrf{A}_2{B}_2u.-\\ {}\kern5.04em -{k}_{dis,Nrf{A}_2{B}_2}\left(Nrf{A}_2{B}_2-{K}_{dis,Nrf{A}_2{B}_2}^{-1}{(NrfAB)}^2\right)-\left({k}_{d,Nrf{A}_2{B}_2}+ kflow\right)Nrf{A}_2{B}_2,\\ {}\frac{dNrf{A}_2{B}_2u}{dt}=-{k}_{dis,Nrf{A}_2{B}_2u}\left(Nrf{A}_2{B}_2u-{K}_{dis,Nrf{A}_2{B}_2u}^{-1}Nrf{A}_2{B}_2\times u\right)-\left({k}_{cat,Nrf{A}_2{B}_2u}+ kflow\right)Nrf{A}_2{B}_2u.\end{array}\right. $$

The first equation in the (1) model describes the total change rate of the nitrite concentration in the chemostat, which is a sum of nitrite inflow and outflow rates into/from the chemostat, the rate of the NrfA, NirC and nitrite enzyme-substrate complexes formation and the rate of the nitrite export from *E. coli* cells. Since *C* is the total cell volume fraction in the chemostat volume, in the first equation rates of processes that occur within an individual cell are multiplied by the *C* value. Other equations describe processes that occur within an individual cell.

The second equation describes the change rate of the intracellular nitrite concentration, and other equations–formation processes of the NrfA and NirB reductases and NirC transporter active forms, transport and catalytic reactions.

### Estimation of the model parameters

The complete list of (1) model parameters and their values used in the calculations are represented in the Appendix: Table 1. The procedure for obtaining parameter values for the *m*_*Nrf*_(*u*), *m*_*Nir*_(*u*) and *U*(1) functions was described above (see subsystems (6) and (7)). In this section we will consider the (1) model parameters, the values of which were obtained on the basis of the existing experimental data on the dynamics of the *E. coli* cell culture growth under glucose-limiting conditions in the flow chemostat, the kinetics of the nitrite utilization by NrfA and NrfB nitrite reductases and parameters of the NirC protein mediated nitrite transport [5–7, 9, 13, 18, 19, 21–24].

Parameters of the protein passage from the cytoplasm to the periplasm under the membrane potential influence were estimated on the basis of the assumption that Nrf enzyme is transported from the cytoplasm to the periplasm as NrfA and NrfB monomer subunits, which are synthesized in the cytoplasm (subsystem (6)). The ratio of the cytoplasmic volume to the periplasmic volume *δ*_*peripl*_ was assessed based on the experimental data, according to which the periplasmic space proportion makes 8–40 % of the whole *E. coli* cell volume [25–27]. On this basis, it is determined that the *δ*_*peripl*_ value may vary in the 2.5–12.5 range. In the model *δ*_*peripl*_ is equal to six (Appendix: Table 1).

When evaluating the degradation rates of NrfА, NrfВ, NirB, NirD, NirC proteins and their complexes, we considered the mean half-life of a pool of *E. coli* cytoplasmic proteins (~2 h) [28] and stability of the periplasmic fraction of protein complexes, which is significantly higher [29].

The rate constant values for the Nrf reductase dimeric and trimeric forms dissociation were estimated based on the experimental data [18].

The rate constant values for the NrfAB, NrfA_2_B_2_, NirC_5_, NirB_2_ and NirB_2_D complexes formation were evaluated on the basis of indirect data on the protein-protein interaction kinetics [30].

The *E. coli* cell culture volume fraction in the chemostat volume was assessed based on the previous data [7], according to which when glucose concentration in the medium is fixated (2.25 mM) and glucose inflow rate is constant, the cell growth rate is given and constant. The cell cycle duration under such conditions is 70 min. Accordingly, the *C* parameter in the (1) model is assumed constant. Considering the described above parameters for the culture development in the chemostat and the rate of glucose uptake by the cells under similar limiting conditions [24], the range of the *C* parameter possible variation was evaluated: 0.0001≤C≤0.002. The *C* = 0.0003 parameter value was taken in the model analysis.

Values of other parameters that have not been evaluated on the basis of experimental data were estimated in the process of model numerical adaptation to the experimental data on the nitrite accumulation in the chemostat [7].

### The model analysis approach

The model analysis approach was selected based on the purpose of the study, which was to explain the kinetic data on the nitrite accumulation in the chemostat according to molecular-genetic mechanisms of nitrite utilization in *E. coli* cell cultured in a flow chemostat.

Since the target experiment performed in [7] consisted of a series of measurements of nitrite equilibrium concentrations, which were established in the chemostat at every given constant nitrite inflow rate, calculations of the positive stationary points were also performed in the (1) model.

Positive stationary point for each given *s* value of the added nitrate was found numerically by solving the system of algebraic equations, which was obtained by equating the right-hand side of the (1) model to zero. The Mathematica program was used for all calculations. Positive solutions found in all calculations were the only ones in a positive change range of the system arguments.

## Results and Discussion

### Assessment of the contribution of mechanisms regulating the activity of enzymes involved in NO_2_ metabolism and transport to the control of NO_2_ intracellular levels

As discussed above, the existing amount of experimental data on *nrfA, nirB* and *nirС* gene expression regulation and nitrite accumulation dynamics during *E. coli* stationary phase growth in the chemostat, presented by Wang et al [1, 7], allowed us to develop a mathematical model of nitrite intracellular utilization and investigate the role of various components of the nitrite utilization system in controlling nitrite intracellular level.

**Fig. 4:**
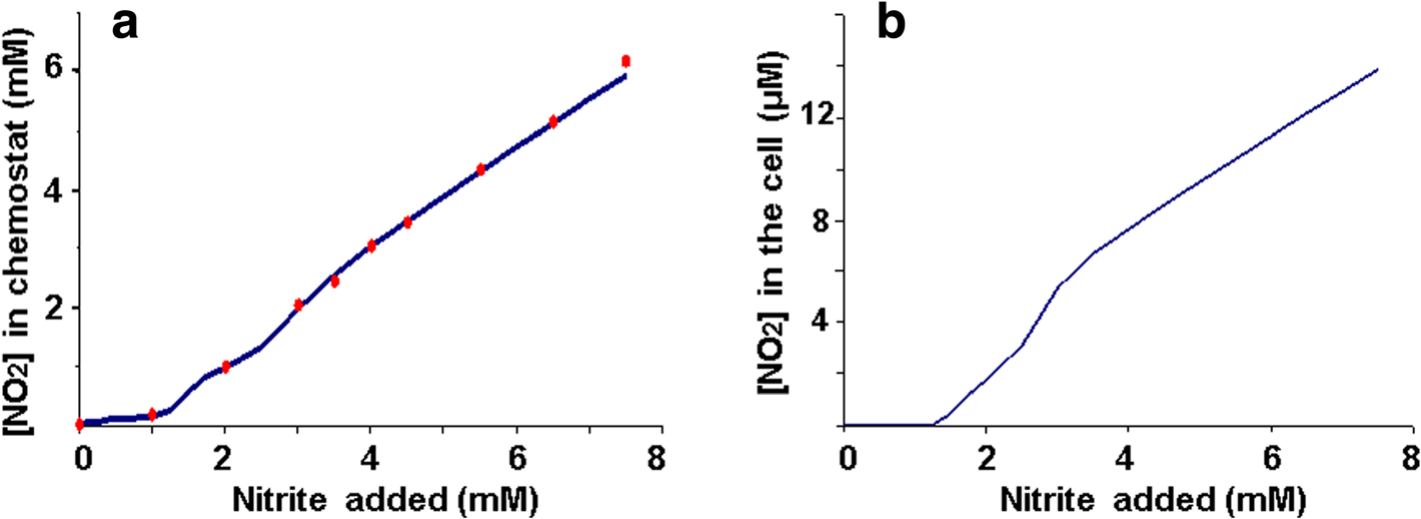
The steady-state nitrite concentration in a chemostat and inside a cell. Curve – (1) model calculation, dots – experimental data from Wang et al. [7], X-axis – added nitrite concentration (mM), Y-axis: **а** – steady-state nitrite concentration in the chemostat (mM), **b** – nitrite concentration in a cell (μM). Parameter values are presented in the Appendix: Table 1

After adapting the model to the experimental data, the (1) model calculation results, presented in Fig. 4, demonstrated a good agreement between theoretical curve and quantitative data on the nitrite accumulation dynamics in a chemostat (Fig. 4a). The maximum value of the intracellular nitrite concentration in the studied range of the added nitrite did not exceed 14 μM (Fig. 4b), which is in qualitative agreement with the experimental data on the maintenance of sufficiently lower, less than 0.1 mM, nitrite concentration in the cell under *E. coli* cell cultures anaerobic growth at 20 mM nitrate, when the nitrite concentration in the growth medium reaches 5 mM [5].

**Fig. 5:**
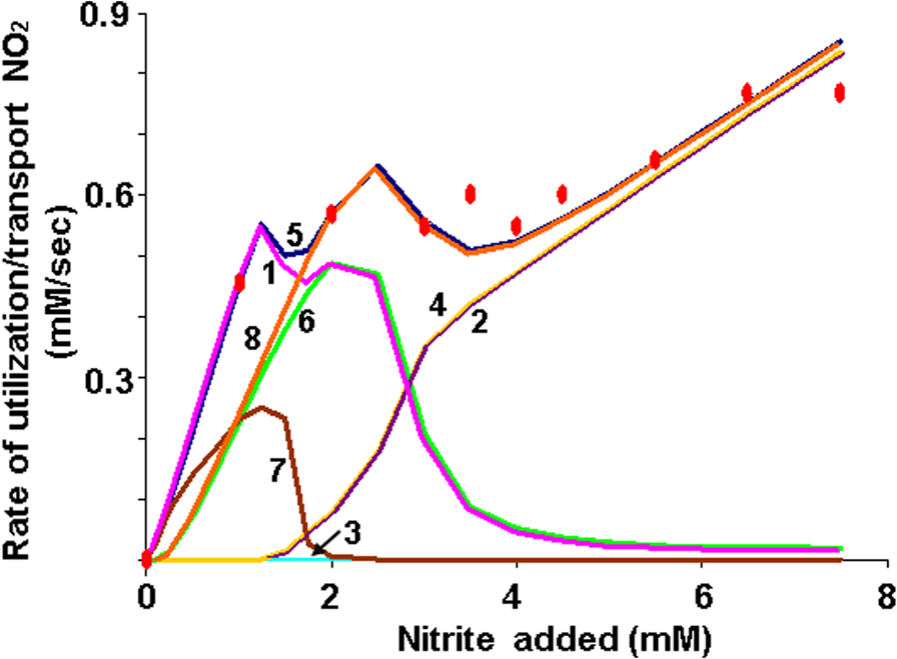
Contribution of the NO_2_ utilization and transport system various components to the nitrite accumulation dynamics. Dots – experimental data on the nitrite utilization by the cell culture population, calculated with the *kflow*(*s-u*)/*C* law based on the data from [7]; curves 1-5 – (1) model calculation; curves 6-8 – (*s*1_−7_) model calculation; curve 1 – the rate of nitrite utilization by the perilasmic NrfA reductase; curve 2 (dashed) – the rate of nitrite import by the NirC transporter; curve 3 – the rate of nitrite export by the NirC transporter, measured in negative units; curve 4 – the rate of nitrite utilization by the cytoplasmic NirB reductase; curve 5 – the total rate of nitrite utilization by NrfA and NirB reductases; curve 6 – the rate of nitrite utilization by the NrfА reductase, implemented in the (*s*1_−7_) model, excluding the effect of the membrane potential (*dU* = 0) on the NirA and NirB proteins diffusion rate *kt*_*Nrf,cp*_ = 0.055 s^−1^; curve 7 – difference between curves 1 and 6; curve 8 – the total rate of nitrite assimilation by the cell, calculated with (*s*1_−7_). Other parameters values are presented in the Appendix: Table 1

Using the (1) model calculations, various components of the nitrite utilization system in *E. coli* cells cultured in a flow chemostat at different concentrations of the added nitrite were evaluated for their contribution to the nitrite accumulation dynamics in the chemostat and inside the cell (Fig. 5, curves 1–5).

We see the periplasmic NrfA reductase (curve 1) and cytoplasmic NirB reductase (curve 4) contributions to the nitrite utilization dynamics in the chemostat (curve 5), the experimental parameters of which were calculated based on the data from [7] and are shown with dots. The NirC transporter activity dynamics in relation to the nitrite import into the cell (curve 2) and its export from the cell (curve 3) was demonstrated.

Analysis of results has shown that the range of the added nitrite concentrations can be divided into two nominal range areas: the area of low values 0 ≤ *s* ≤ ~1.25 mM and the area of high values *s* > ~3.5 mM, in which nitrite is utilized only by the periplasmic or the cytoplasmic nitrite reductase, respectively. This is due to the functional peculiarities of the *nrf* and *nir* operons, encoding these enzymes, the activity of which depends on the nitrite concentration [1].

Let us consider the area of high values of the added to the chemostat nitrite (>3.5 mM). In this range there is a high nitrite processing efficiency of the NirB reductase (curve 4), which is sufficient for describing the experimentally observed nitrite utilization rate in *E. coli* cells (dots), and low nitrate processing efficiency of the NrfA reductase (curve 1), which is consistent with the expression dynamics of encoding these enzymes operons [1].

Moreover, according to the model, the nitrite import rate (curve 2) strictly corresponds to the rate of its processing by the NirB reductase (curve 4), indicating the close association of the intracellular nitrite utilization system with the transport system of nitrite import into the cell at high substrate levels, and is a result of the arrangement of genes encoding their structure in one operon.

However, as seen in Fig. 5, with the parameter values taken (Appendix: Table 1), nitrite export from the cell into the medium by the NirC transporter is practically absent (curve 3). Such property of the model, in our opinion, is a result of some parameter values taken during calculations (Appendix: Table 1) and is associated with the transport system, the evaluation of which was carried out based on the *S. typhimurium* NirC catalytic property data [31].

According to the [31], *S. typhimurium* NirC is active in the millimolar range of the nitrite concentration. These data transfer to the *E. coli* cell system (*Km*_*NirC,in*_ = *Km*_*NirC,out*_ = 1 mM) puts practically no restrictions on the nitrite import process as steady-state nitrite concentration in the chemostat in the range of added nitrite > 3.5 mM is higher than 2.5 mM.

However, when using those parameters of nitrite utilization in the cell by the cytoplasmic NirB reductase, taken from [21], in the model calculations, when *s* > 3.5 mM, the intracellular nitrite concentration was in the range of 6 to 15 μM (Fig. 4b). It is not surprising that at such nitrite concentrations in the cell the export activity of the enzyme is practically absent.

It should be noted that the parameters of the nitrite export from the cell (in particular, the *Km*_*NirC*,*out*_ value) indirectly indicate the concentration threshold of nitrite toxicity for cells. Therefore, if the value *Km*_*NirC,out*_ = 1 mM is valid for *E. coli* cells, the intracellular nitrite becomes toxic for *E. coli* cells only at concentrations of about 1 mM.

Based on this, under experimental conditions, implemented in [7], the cell functions far from the toxicity threshold and has no need for nitrite export from the cell. However, experimental data indicate the significant NirC transporter export activity [6], so the need for *Km*_*NirC*,*out*_ =1мМ value to explain the data on the nitrite accumulation in the chemostat [7] in the range of high added nitrite concentration remains an open question. We will consider this issue below.

Let us now consider the range of low added nitrite concentrations (s < 1.25 mM). As stated above, in this range, the inflowing nitrite is utilized only by the periplasmic NrfA reductase (Fig. 5a, when *s* < 1.25 curve 1 matches curve 5).

High Nrf reductase activity is ensured by the combined effect, which is achieved through the activation of *nrf* operon expression (Fig. 2a), the *de novo* synthesis of NrfA and NrfB proteins, and through the increased rate of NrfA and NrfB proteins passage from the cytoplasm to the periplasm as a result of the membrane potential *U* formation (Fig. 3).

Let us consider the potential contribution of each mechanism in the formation of the nitrite utilization rate in *E. coli* cells cultured in the chemostat.

### Contribution evaluation of the genetic component of the NrfA periplasmic reductase activity regulation mechanism to the NO_2_ utilization in *E. coli* cells

To assess the contribution of the Nrf reductase encoding *nrf* operon expression regulating mechanisms to the nitrite utilization in the chemostat at micromolar concentrations of the substrate we explored the (1) model functioning dynamics, ignoring mechanisms of membrane potential formation.

To do this, we put value *d*_*U*_ = 0, excluding subsystem 7 from the model (hereinafter, we denote this model version as (*s*1_−7_). In this version of the model, to achieve best agreement between calculations and experimental data, we had to increase the rate constant value of the constitutive NrfA and NrfB proteins transport from the cytoplasm to the periplasm, compared to the baseline, by 5.5 times (*kt*_*Nrf*,*cp*_ = 0.055 s^−1^). The results are shown in Fig. 5 (curves 6–8).

It is well seen that the NrfA reductase activity obtained by calculating the (*s*1_−7_) model, in which the NrfA activity is determined only by the nitrite-dependent genetic mechanisms of the *nrf* operon expression regulation (curve 6) at 1 mM point, is significantly lower than that obtained by calculating the total (1) model (curve 1). The difference between these activities (curve 7) is a bell curve, which corresponds to the added activity, which provides an adequate description of the experimental data on nitrite utilization in the chemostat by the (*s*1) model (curve 5) as shown in Fig. 5 with dots. This added activity for describing the same data is missing in the (*s*1_−7_) model (Fig. 5, curve 8). Thus, an adequate description of the nitrite accumulation kinetics in the chemostat in the micromolar range of the added nitrite concentration (<1 mM) cannot be achieved on the basis of the genetic data of nitrite-dependent *nrf* operon expression activity [7].

From here, we conclude that membrane potential plays an important role in the nitrite utilization dynamics in the micromolar range of the substrate concentration in the chemostat. Above, we have given calculations that showed that it is sufficient to consider the membrane potential impact on the rate of the enzyme redistribution between the cytoplasm and the periplasm for explaining the experimental data.

However, since there is evidence of other possibilities of membrane potential influence on the NrfA reductase activity, it is necessary to consider them as alternative mechanisms for explaining the data on nitrite consumption in the chemostat. These mechanisms are discussed in the next section.

### Mechanisms of influence of the membrane potential on the periplasmic NrfA reductase activity

We should note that, according to the existing data, membrane potential plays a significant role in the formation of catalytically active Nrf enzyme molecule, affecting its passage into the periplasm, its stability and catalytic properties. It is known that protein passage into the periplasm is initiated by the electric membrane potential advent as a result of the activity of respiratory enzymes involved in the proton gradient formation [32]. Nrf reductase refers specifically to such enzymes and nitrite is an electron acceptor [33]. Proteins passage into the periplasm affects their stability [29]. Enzyme assembling and correct orientation in the periplasmic space is also dependent on the presence of membrane potential [32, 34]. Moreover, it has been shown that Michaelis constant for the Nrf enzyme is dependent on the membrane potential value [22], and the membrane potential value is dependent on the nitrite concentration [16].

To answer the question of which of the above mechanisms is essential to explain the experimental data, we evaluated the each mechanism contribution to the nitrite utilization at low added nitrite levels in the chemostat.

First, we tested if the enzyme catalytic properties change under the influence of the membrane potential can affect the nitrite utilization kinetics in the chemostat.

It is known that Michaelis constant for the NrfA enzyme varies depending on the membrane potential (*K*_*m,nrf*_ = 0.03 mM at -0.4 V and 0.012 mM at -0.3 V) [11].

Analysis of the (s1_−7_) model have shown that the *K*_*m,nrf*_ value change by an order of magnitude or more, that goes beyond the experimentally observed differences [11], leads to minor changes (in the redistribution of a few percent) of the NrfA mediated nitrite utilization rate in the examined range and cannot be a source of additional enzyme activity at low nitrite concentrations, sufficient to explain the experiments [1].

Introduction to the (*s*1_−7_) model a positive non-linear relationship between the NrfA reductase catalytic activity and nitrite concentration in the medium allows to achieve the desired effect, however, this hypothesis is not supported by the data on a simple reaction kinetics described by the Michaelis-Menten equation [11]. Regarding the membrane potential impact on the rate of the enzyme passage into the periplasm and catalytically active enzyme molecule assembly, the consideration of this process is consistent with experimental data and, according to the (1) model analysis presented above, is sufficient to describe the experimental data on nitrite accumulation dynamics in the chemostat [7].

It should be noted that passage of enzyme subunits into the periplasm under the membrane potential influence is also accompanied by an increase in their local concentrations and stability. How important are these processes to explain the experimental data?

The local concentration coefficient of variation is equal to the ratio of cytoplasmic volume to periplasmic volume, which, based on the experimental data [25–27], is equal to six in the model (*δ*_*peripl*_ = 6).

In essence, the mechanism of membrane potential is to increase the rate of the NrfA and NrfB proteins transport from the cytoplasm to the periplasm and provide the accumulation of proteins synthesized in the cytoplasm, in a smaller volume of periplasm. The *δ*_*peripl*_ = 6 ratio used in a model is sufficient to ensure that the concentration of Nrf enzyme in the periplasm reached a level ensuring adequate nitrite consumption rate in the chemostat, meeting the experimental data. Note that the enzyme concentration increase is equivalent to the enzyme catalytic activity value increase (which we previously ignored as it is not backed by experimental data), as during the catalytic process the absolute reaction rate is determined by multiplying the catalytic constants by the concentration of the enzyme active molecules.

Theoretically, it is also possible that the increase of protein stability in the periplasm by almost an order of magnitude [29], which is also considered in the model, may be a crucial factor. However, according to the calculations, this factor is of secondary importance, and it is not required to explain the experimental data.

Thus, the model analysis demonstrates that membrane potential significantly contributes to the regulation of the periplasmic NrfA reductase activity and it must be considered in order to explain the nitrite utilization dynamics at substrate concentrations <1 mM. The most probable mechanism is the rate change of NrfA and NrfB proteins passage from the cytoplasm to the periplasm, depending on the membrane potential value.

In light of these theoretical results, attention is drawn to the fact that both mechanisms: the nitrite-dependent membrane potential change and the nitrite-dependent effectiveness of *nrf* operon expression, have qualitatively identical unimodal activity curves (Figs. 2a and 3). As a result, the necessity of addressing both mechanisms to explain the experimentally observed nitrite accumulation curve in the low range of the added nitrite concentration emerges. This question is discussed in the next section.

### On the functional redundancy of the NO_2_ utilization mechanisms in *E. coli* cell

In this section, we analyze the possibility of (1) model functional redundancy in the utilization mechanisms and related issues. The essence of the matter derives from the fact that subsystem (1)–(11), described above and including a certain number of genetic, transport, complex-forming, and enzymatic reactions, make up the structural basis of the model. We used two approaches to describe the subsystems. Regulation of the *nrf* and *nir* operons expression efficiency, as well as the membrane potential, we described with phenomenological functions of the class of generalized Hill functions [8]. Other processes (formation of the protein complexes, enzymatic reactions, transport from the cytoplasm to the periplasm, degradation of proteins and their complexes) were described with non-equilibrium biochemical reactions. Meaning the right-hand side of (1) system includes members that describe both direct and reverse non-equilibrium processes. At the same time, to describe the dynamic characteristics of the nitrite accumulation in the chemostat, the (1) system equilibrium state has to be calculated. It is well known from the general theory of dynamical systems that the equilibrium state specific the value is not determined by the values of the parameters, but by values of their specific combinations, the number of which is usually less than the number of the parameters themselves. The simplest example is the bimolecular reaction. To describe the reaction far from equilibrium, it is necessary to know the rate constant values of the forward and reverse reactions. However, at equilibrium, it is sufficient to know the value of one equilibrium constant, which is the ratio of non-equilibrium constants. As a result, we get a traditional problem of determining the degree of functional and parametric redundancy of mechanisms reflected in (1), which are necessary to describe the nitrite consumption kinetics in the chemostat.

In this context, we consider the redundancy issue of mechanisms, by which the nitrite accumulation kinetics in the chemostat at concentrations of the added nitrite *s* < 2 mM is explained in the model.

As stated above, the apparent similarity of the curves describing the *nrf* operon expression and membrane potential influence on the rate of the NrfA and NrfB proteins redistribution in relation to the added nitrite concentration (Figs. 2a and 3) suggest that in order to explain the experimental data from [7] it is sufficient to use only one of these mechanisms and the other can be omitted. Is it so?

As for the need to consider the membrane potential, we showed above that without considering the influence of membrane-dependent potential on the rate of the NrfA and NrfB proteins redistribution between the cytoplasm and the periplasm it is impossible to adequately describe the experimental data on nitrite utilization in the chemostat [7]. This output, as shown above, is considerably based on the data on nitrite-dependent genetic regulation of *nrf* operon expression (see Fig. 5). However, let’s assume that *nrf* operon is constitutively expressed. It appears that for this version of (1) model it is not difficult to pick up a set of parameter values (for example: *m*_*Nrf*_(*u*) ≡ 1, *m*_*Nir*_(*u*) ≡ 0, *k*_*s,NrfA*_ = *k*_*s,NrfB*_ = 0.0000045 mM/s, *K*_*Nrf1*_ = 0.11, *h*_*Nrf,*1_ = 2, *δ*_*Nrf,*2_ = 0.0375, *K*_*Nr,f*2_ = 0.6, *h*_*Nrf,*2_ = 8, *ω*_2_ = 0.005, *K*_*Nrf,*3_ = 2.2, *h*_*Nrf,*3_ = 15, values of other parameters are unchanged and presented in the Appendix: Table 1), for which the model will adequately describe the nitrite accumulation curve.

That is, the data on the genetic regulation of the *nrf* operon expression are redundant for explaining the nitrite accumulation curve in relation to the added nitrite concentration. It is sufficient to assume the *nrf* operon constitutive expression and take into account the membrane potential impact on the NirA and NirB proteins redistribution between the cytoplasm and the periplasm. However, this conclusion is theoretical, since the native *E. coli* cells reveal a strong nitrite-dependent regulation of the *nrf* operon expression [1]. And, nevertheless, the revealed redundancy raises important theoretical question of evolutionary expediency of such a complex mechanism of *nrf* operon expression regulation in *E. coli* cells: because the cell could easily utilize nitrite in simpler and more economical way! This issue requires further study and more detailed analysis.

The second issue considered in this section, is the redundancy of the nitrite export mechanism. It is indeed known that *E. coli* cells have a certain threshold value of the intracellular nitrite concentration, above which it becomes toxic to the bacteria. It is believed that in this case the cell has a protective system based on the NirC nitrite export activity, which is basic during nitrite breathing [6]. However, the parameters of such activity were not measured. In the model, we used values of the parameters that have been measured for the *S. typhimurium* NirC transporter.

**Fig. 6:**
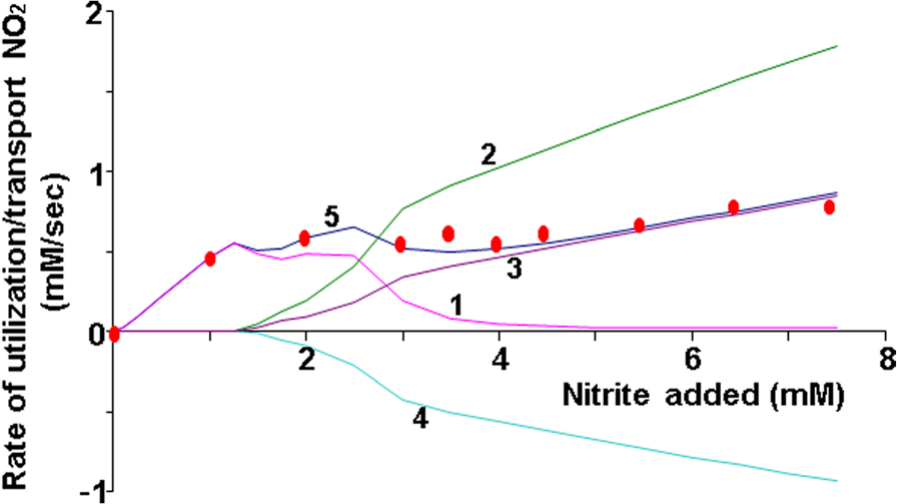
Nitrite utilization and transport rate in relation to the concentration of the added nitrite. Dots – the rate of nitrite consumption by the cell, calculated based on the experimental data from [7]; curve 1 – the rate of nitrite utilization by the periplasmic NrfA reductase; curve 2 – the rate of nitrite import into the cell by the NirC transporter; curve 3 – the rate of nitrite export from the cell by the NirC transporter, measured in negative units; curve 4 – the rate of nitrite utilization by the cytoplasmic NirB reductase; curve 5 – the total rate of nitrite utilization by NrfA and NirB reductases. Calculations performed with the (1) model version, in which *K*_*М,NirC,out*_ = 0.003 mМ, *k*_*s,NirC,*_0 = 0.00011 mM/s; other parameter values are from the Appendix: Table 1

But then, it implies that (a) the nitrite concentration toxic for *E. coli* cells is comparable to millimolar concentrations, and (b) in the (1) model nitrite export from cells is practically absent (Fig. 5, curve 3). However, there is reason to believe that in *E. coli* cells nitrite toxic concentration is in the micromolar range [5], i.e., Michaelis constant for the NirC mediated nitrite export can be much smaller than the one given in Appendix: Table 1. On this basis, we have considered a model version, in which the value of the Michaelis constant for the NirC mediated nitrite export from the cell is 300 times smaller: *K*_*M*,*NirC*,*out*_ = 0.003μМ. The corresponding calculation is presented in Fig. 6.

Calculations have shown that such parametric version of the model does not impair the data reproduction quality. At the same time, under a given set of parameter values there is a high rate of nitrite export from the cell into the chemostat (Fig. 6, curve 5). Consequently, the ratio of NirC transporter import activity functional parameter to its export activity functional parameter is a free parameter in the (*s*1) model, and to determine its value, additional information that is not available in the scientific literature is needed.

In conclusion, the nitrite concentration curve at different values of the added nitrite concentration, shown in Fig. 4b, in a sense, is nominal. It is easy to choose sets of parameters for which the model calculations of the intracellular nitrite concentration will be different from the above calculation in orders of magnitude, and, at the same time, the accuracy of the nitrite accumulation approximation curve will not be altered (for example: *ks*_*NirC*_ = 0.00011 mM/s, ${K}_{dis,Nir{C}_5w}$ = 3 μM, values of other parameters are unchanged and presented in the Appendix: Table 1), so this characteristic is also free in the model and for its more accurate characterization it is necessary to have additional data, which is not yet available.

## Conclusion

In the present work we introduce the model of nitrite utilization in *E. coli* cells cultured in the flow chemostat. The parameter values were chosen in such a way that the model adequately describes the experimental curve of nitrite accumulation in the chemostat [7]. The model analysis revealed the existence of two ranges of the added NO_2_ concentration: low (*s* < 1.25 mM) and high (*s* > 3.5 mM). According to the model, in the high concentration range the nitrite is substantially utilized by the nitrite transport/utilization system (NirC transporter/NirB reductase). In this range, the nitrite-dependent genetic regulation of the *nir* operon expression is sufficient for explaining experimental data on the nitrite accumulation in the chemostat [7].

On the contrary, in the low concentration range of the added nitrite the known genetic mechanisms of the *nrf* operon expression are not enough for explaining experimental data on the nitrite accumulation in the chemostat [7]. Analysis of different hypothesis has shown that the most probable additional mechanism of the periplasmic Nrf reductase activity regulation in the micromolar concentration range of the added nitrite, that allows co-ordination of physiological and genetic data [1, 7], is a local enzyme concentration change due to its passage from the cytoplasm to the periplasm under the influence of nitrite concentration-dependent membrane potential. In the framework of our model, the potential-dependent mechanism of the NrfA and NrfB proteins redistribution between cytoplasm and periplasm is the essential element required to explain observed nitrite accumulation dynamics in the chemostat in the low concentration range of the substrate.

Analysis of different nitrite utilization and transport mechanisms contribution to the nitrite accumulation in the chemostat and inside the cell revealed some redundancy of molecular-genetic mechanisms of NrfA, NrfB reductases and NirC transporter activities regulation. Thus, to describe the nitrite accumulation curve in chemostat in the entire concentration range of the added nitrite it is sufficient to assume a constitutive *nrf* operon expression, whereas the NirC transporter and NirB nitrite reductase can be ignored.

In the high concentration range of the added nitrite the ratio of NirC transporter import activity functional parameter to its export activity functional parameter as well as intracellular nitrite concentration are free parameters of the model and may vary over a wide range without affecting the nitrite accumulation curve description accuracy of the model.

## Appendix

**Table 1:** List of the model parameters

Parameter	Parameter name	Parameter value	Sub system	Estimation source
*k*_*flow*_	the flow rate constant	1.65 · 10^−4^ s^−1^	(1)	[1]
δ_*nrf,*1_	parameters of the *m*_*nrf*_ generalized function that describes the *nrf* operon activity changes in relation to the nitrite concentration established in the chemostat	60	(6)	[1, 35]
*K*_*nrf*,1_		0.36 mM		
*h*_*nrf*,1_		1.3		
δ_*nrf,*2_		0.16		
*K*_*nrf*,2_		1.7 mM		
*h*_*nrf*,2_		3.0		
*ks*_*Nrf*_	the maximum specific rate of the NrfA and NrfB proteins synthesis	1.13 10^−5^ mM/s	(6)	[*]^*b*^
*ks*_*NirC*_	the maximum specific rate of the NirC potein synthesis	7.3 · 10^−5^ mM/s	(6)	
$$ \begin{array}{l}k{s}_{NirB}=\\ {}=k{s}_{NirD}\end{array} $$	the maximum specific rate of the NirB and NirD proteins synthesis	$$ \begin{array}{l}k{s}_{NirB}=\\ {}=k{s}_{NirC}/5\end{array} $$	(6)	
*k*_*d*,*Nrf*_	the rate constant for the NrfA and NrfB monomers degradation in cytoplasm	9.6 10^−5^ s^−1^	(11)	[29]
*kt*_*Nrf*,*cp*_	the rate constant for the NrfA and NrfB passage into the periplasm	0.01 s ^–1^	(7)	[*]^*b*^
*kt*_*Nrf*,*pc*_	the rate constant for the NrfA and NrfB transport into the cytoplasm	10 s ^–1^	(7)	
*k*_*dis*,*NrfAB*_	the rate constant for the NrfAB dimer dissociation into subunits	10 s ^–1^	(9)	
*K*_*dis*,*NrfAB*_	the equilibrium dissociation constant for the NrfAB dimer	0.00004 mM	(9)	[18]
${k}_{dis,Nrf{A}_2{B}_2}$	the rate constant for the Nrf(AB)_2_ dissociation into two NrfAB dimer proteins	10 s ^–1^	(9)	
${K}_{dis,Nrf{A}_2{B}_2}$	the equilibrium dissociation constant for the NrfA_2_B_2_ tetramer	0.004 mM	(9)	
*δ*_*peripl*_	the constant for the cytoplasmic volume to periplasmic volume ratio	6	(7)	[25]
*d*_*U*_	parameters of the *U* generalized function that describes membrane potential relative value in relation to the added NO_2_	3	(7)	[16]
*δ*		0.015		
*K*_*pmf*,1_		0.115 mM		
*h*_*pmf*,1_		2		
*K*_*pmf*,2_		0.83 mM		
*h*_*pmf*,2_		17		
*ω*_1_, *ω*_2_^с^		0^с^	(7)	[*]^*b*^
*K*_*pmf*,3_^с^		2.2		
*h*_*pmf*,3_^с^		15		
*k*_*d*,*NrfA*_	the rate constant for the NrfA monomer degradation in cytoplasm	9.6 10^−6^ sec ^–1^	(11)	[29]
*k*_*d*,*NrfA*_	the rate constant for the NrfA monomer degradation in the periplasm			
*k*_*d*,*NrfAB*_	the rate constant for the NrfAB degradation			
${k}_{d,Nrf{A}_2{B}_2}$	the rate constant for the NrfA_2_B_2_ degradation			
${k}_{cat,Nrf{A}_2{B}_2u}$	the rate constant for the NrfA reductase turnover	700 s ^–1^	(2)	[9]
${K}_{dis,Nrf{A}_2{B}_2u}$	the NrfA reductase Michaelis constant for nitrite	0.03 mM		[10]
δ_*nir,*1_	parameters of the *m*_*nir*_ generalized function that describes the *nir* operon activity changes in relation to the nitrite concentration established in the chemostat	7.86	(6)	[1, 35]
*K*_*nir*,1_		1.0 mM		
*h*_*nir*,1_		2.0		
δ_*nir,*2_		5.76		
*K*_*nir*,2_		1.7 mM		
*h*_*nir*,2_		8.3		
${K}_{dis,Nir{B}_2}$	the equilibrium dissociation constant for the NirB_2_ association reaction	0.002 mM	(10)	[*]^*b*^
${K}_{dis,Nir{B}_2D}$	the equilibrium dissociation constant for the NirB_2_D trimer association reaction	0.002 mM	(10)	
${k}_{cat,Nir{B}_2Dw}$	the rate constant for the NirB enzyme catalytic turnover	1100.0 s ^–1^	(5)	[*]^*b*^
${K}_{dis,Nir{B}_2Dw}$	assumed to be equal the NirB reductase Michaelis constant	0.006 mM		[21]
${K}_{dis,Nir{C}_5}$	the rate constant for the NirC_5_ dissociation	0.0475 mM	(8)	[*]^*b*^
*k*_*cat*,*NirCin*_	the rate constant for nitrite import by the NirC protein	100 s ^–1^	(3)	[*]^*b*^
${K}_{dis,Nir{C}_5u}$	assumed to be equal the Michaelis constant for nitrite import by the NirC protein	1 mM	(3)	[31]
*k*_*cat*,*NirCout*_	the rate constant for the nitrite export by the NirC protein	1000 s^−1^	(4)	[*]^*b*^
${K}_{dis,Nir{C}_5w}$	assumed to be equal the Michaelis constant for the nitrite export by the NirC protein	1 mM	(4)	[31]
*k*_*d,NirC*_	the rate constant for the NirC monomer degradation	0.0011 s^−1^	(11)	[*]^*b*^
*k*_*d,NirC5*_	the rate constant for the NirC_5_ pentamer degradation	0.00011 s^−1^		
*k*_*d,NirB*_	the rate constant for the NirВ monomer degradation	0.00011 s^−1^		
*k*_*d,NirD*_	the rate constant for the NirD monomer degradation	0.00011 s^−1^		
*k*_*d,NirB2*_	the rate constant for the NirВ_2_ dimer degradation	0.000011 s^−1^		
*k*_*d,NirB2D*_	the rate constant for the NirВ_2_D dimer degradation	0.000011 sec^−1^		
*C*	cell volume relative fraction in chemostat	0.0003	(2, 3)	[7, 29]

^*a*^ – mM (millimole/litre), sec (second), if dimension is not specified, the quantity is dimensionless

^*b*^ – parameter value was estimated based on the experimental data

^c^ – members, before which the *ω*_1_ and *ω*_2_ coefficients are standing in the membrane potential formula, are zeroed in (1) model, the formula used in the model calculation is with *nrf* operon constitutive expression and enabled NirC and NirB enzymes functions
